# Supplementary figures and images for: Sex and circadian regulation of metabolic demands in the rat kidney: A modeling analysis
Source: PLoS One. 2024 Jul 17;19(7):e0293419. doi: 10.1371/journal.pone.0293419 (PMC11253979; doi:10.1371/journal.pone.0293419)

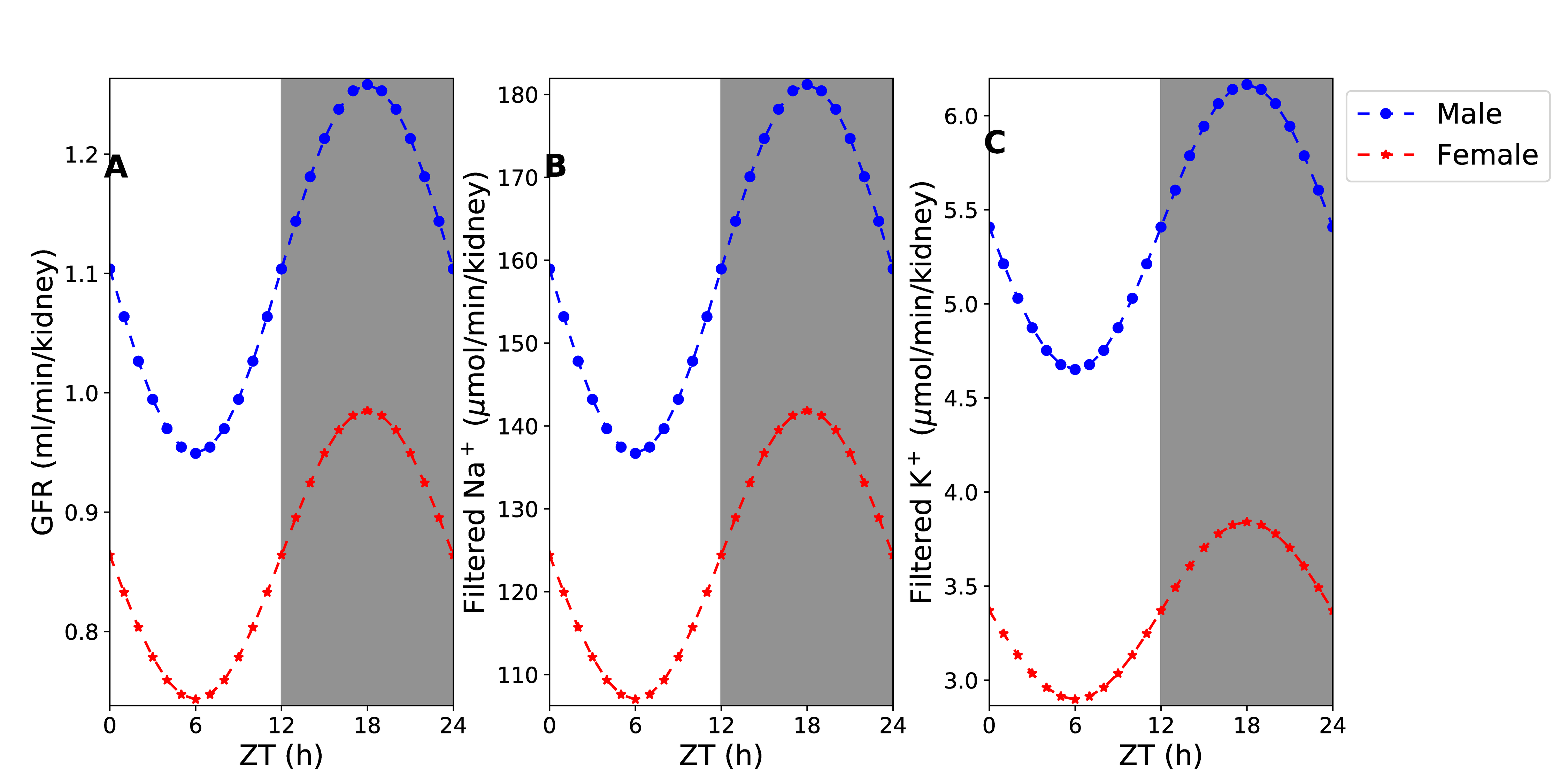

Supplement: S1 Fig — Time profiles of (A) glomerular filtration rate (GFR), (B) filtered sodium load, and (C) filtered potassium load. (TIF) [file pone.0293419.s001.tif]

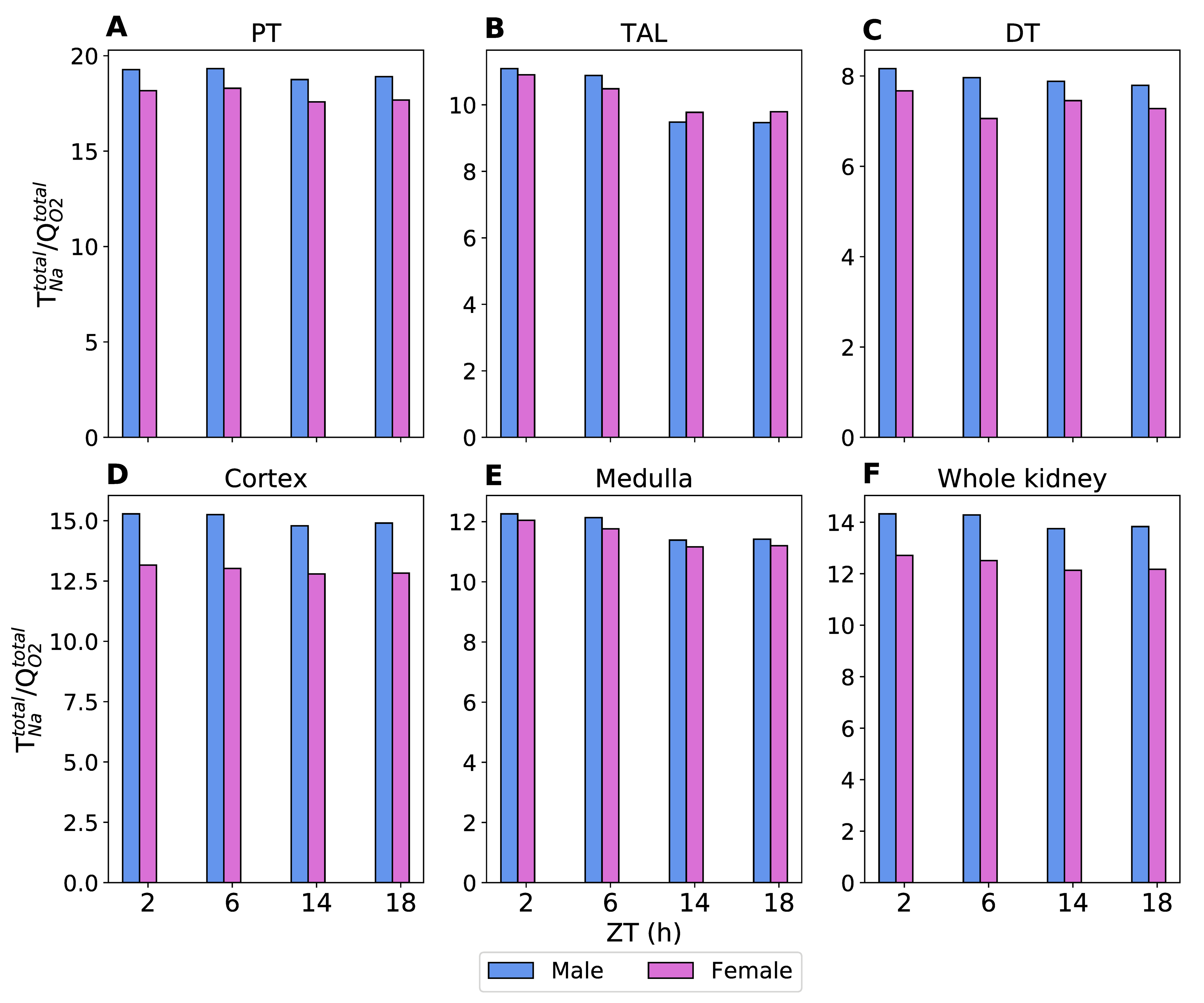

Supplement: S2 Fig — Predicted oxygen utilization efficiency in (A, B, C) the proximal tubules (PT), thick ascending limbs (TAL), and distal tubules (DT); and (D, E, F) the cortical segments, medullary segments, and whole kidney of male and female rats at zeitgeber times 2, 6, 14 and 18 h. The values are given per kidney. (TIF) [file pone.0293419.s002.tif]

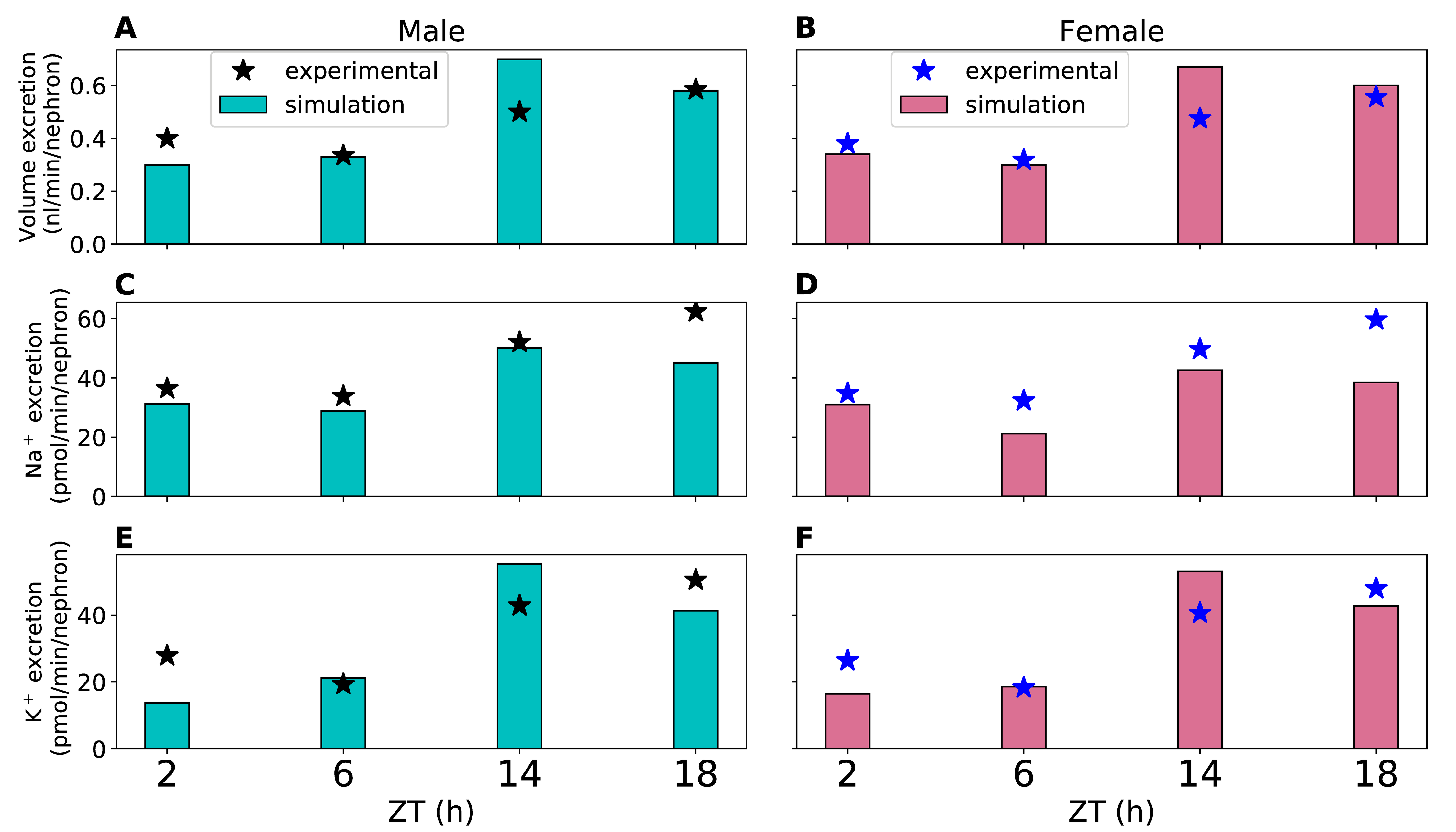

Supplement: S3 Fig — Experimental and simulated volume (A, B), Na+ (C, D), and K+ (E, F) excretion rates in male and female rats at zeitgeber times 2, 6, 14, and 18 h. Circadian oscillations in selected transporter activities were fitted so that the predicted excretion rates are in sufficient agreement with the experimental values. (TIF) [file pone.0293419.s003.tif]
